# Automated fastener (Core-Knot) versus manually tied knots in patients undergoing aortic valve replacement: Impact on cross-clamp time and short-term echocardiographic results

**DOI:** 10.1097/MD.0000000000011657

**Published:** 2018-08-03

**Authors:** Dan Loberman, Rephael Mohr, Paul A. Pirundini, Farhang Yazdchi, Daniel Rinewalt, Tomer Ziv-Baran

**Affiliations:** a Division of Cardiac Surgery, Brigham & Women's Hospital/Cape Cod Hospital, Harvard Medical School, Boston, MA; b Department of Epidemiology and Preventive Medicine, School of Public Health, Sackler Faculty of Medicine, Tel Aviv University, Tel Aviv, Israel.

**Keywords:** aortic valve replacement, cardiac surgery, Core-Knot

## Abstract

The Core-Knot device is an automatic fastener used mainly in minimally invasive heart valve surgery procedures, to facilitate knot tying. The purpose of this report is to compare ischemic time and outcomes of surgical aortic valve replacements (SAVRs) utilizing the Core-Knot device compared with manually tied knots.

Between January, 2014 and December, 2016, 119 patients underwent SAVR in Cape Cod Hospital. We compared patient's characteristics, cross-clamp time, and outcomes of 75 patients who underwent SAVR using Core-Knot to those of 44 operated using manually tied knots.

Patient characteristics were similar between groups. Patients in the Core-Knot group had higher preoperative aortic valve area and higher ejection fraction. The use of Core-Knot was associated with reduced aortic cross-clamp time (median 70 vs 84 minutes; *P* < .001). Patients undergoing SAVR using Core-Knot were less likely to have postoperative aortic regurgitation (*P* < .001). Early mortality, and also the rates of early adverse events (including all cardiac, neurologic, and renal complications), and the immediate postprocedure echo findings were similar in the 2 groups. In multivariate analysis, the use of Core-Knot was associated with reduced postoperative mean gradient across the aortic valve and reduced occurrence of postoperative aortic regurgitation. Older age and larger valve size were other predictors of reduced postoperative mean gradients.

The use of an automatic fastener (Core-Knot) in surgical aortic valve replacement cases reduce aortic cross-clamp time and help eliminate postoperative paravalvular aortic regurgitation.

## Abstract 2

Supplemental Digital Content is available in the text

## 1 Introduction

**Figure 1:**
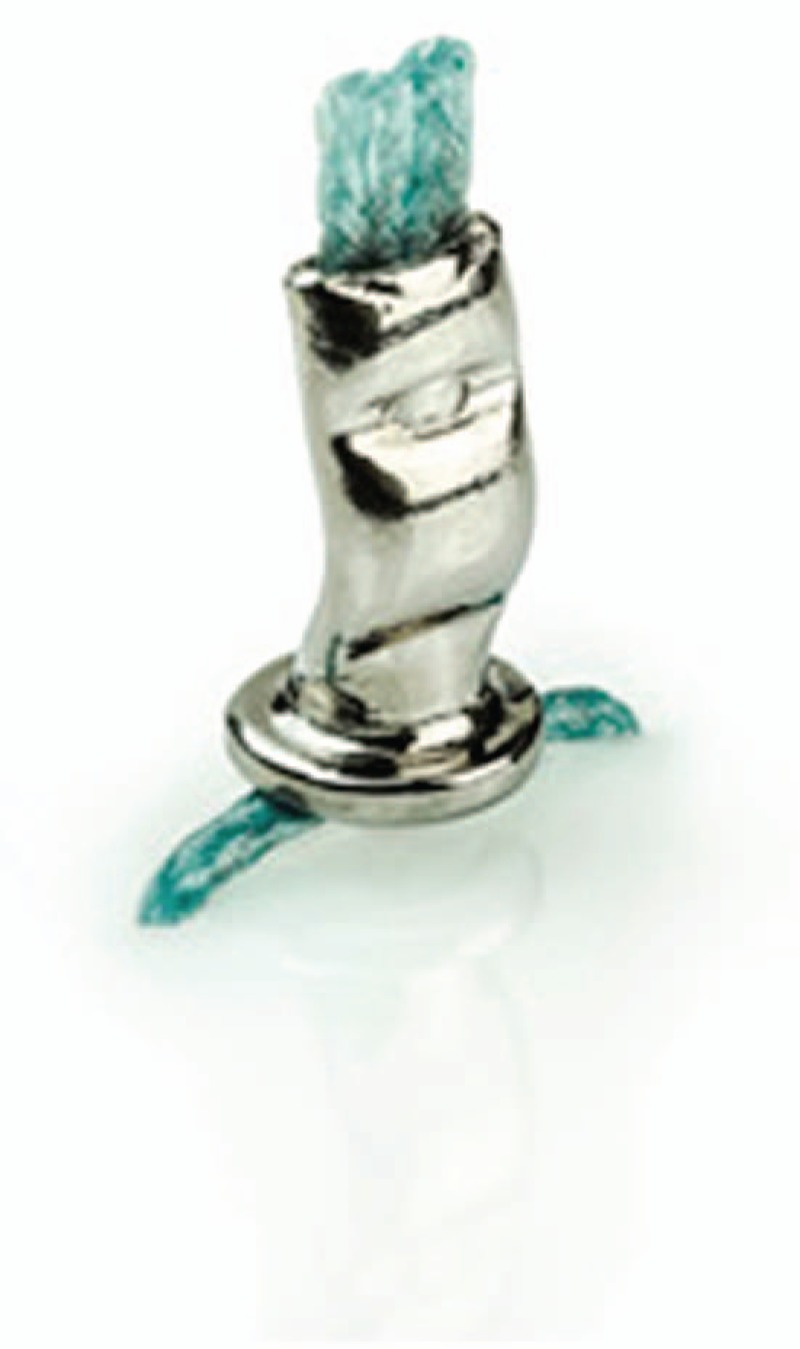
Core-Knot fastener.

Surgical replacement of the aortic valve (SAVR) reduces symptoms and improves survival in patients with severe aortic stenosis.^[1,2]^ Aortic valve surgery case numbers has risen more than 60% since 2012. However, the increase in annual totals is mainly related to the rise in transcatheter aortic-valve replacement (TAVR) procedures performed.^[3]^ The number of SAVR procedures is expected to decrease significantly due to the popularization of the TAVR which is a good and less invasive alternative in certain populations.^[3–5]^ TAVR performed in experienced centers is noninferior to surgery with respect to death or stroke at 2 years.^[6]^ However, occurrences of postoperative aortic insufficiency and atrioventricular (AV) blocks are still higher than the average rates of these complications after SAVR.^[3]^ Reduction of operative times after procedures performed using these approaches can further improve outcome. Significant reduction of operative times after valve surgery was reported with the use of the Core-Knot device [Figure 1, video].^[7]^ Furthermore, the recovery period after SAVR may be shortened with the use of partial sternotomy or mini-thoracotomy.^[8]^ The use of the Core-Knot device may facilitate these minimally invasive approaches.

The purpose of this study was to evaluate ischemic operative time and short-term outcomes of patients undergoing SAVR using the Core-Knot device, and to compare them to those of patients undergoing SAVR using manually tied knots.

## 2 Patients and methods

This is a historical cohort study of all patients who underwent SAVR at Cape Cod Hospital (CCH) between January, 2014 and December, 2016. Manually tied SAVRs were performed until December 10, 2014, and from that day all SAVRs were performed using automatic fasteners (the Core-Knot technique). All SAVR procedures were performed by experience attending surgeons. Preoperative data and early (30 days) outcomes were obtained from review of medical records. Preoperative data included age, sex, and Society of Thoracic Surgeons (STS)-probability of mortality. Operative data included cardiopulmonary bypass time and aortic cross-clamp time. Postoperative data included mean postoperative aortic gradient and degree of aortic and mitral and tricuspid regurgitation. Data on 30-day mortality and postoperative strokes were collected using the CCH medical records data. The study was approved by the Institutional Review Board of the Cape Cod Hospital. Informed consent was waived.

During the study period, 119 patients with aortic stenosis underwent isolated SAVR in CCH. We compared patient characteristics and procedure outcomes of 75 SAVR patients who underwent the procedure using Core-Knot, to those of 44 SAVR patients operated using manually tied knots. Follow-up information was obtained by accessing data from the CCH records.

### 2.1 Definitions

Baseline patient characteristics and in-hospital outcomes were collected according to the STS Adult Cardiac Surgery Database (Data Collection Form Version 2.81-April 23, 2015).^[9]^

### 2.2 Statistical analysis

Categorical variables were expressed as number and percentages. Distribution of continuous variables was assessed using histogram and Q-Q plot. Continuous variables were described using median and interquartile range (IQR). Categorical variables were compared using chi-square test or Fisher exact test, and continuous variables using independent-samples *t* test or Mann–Whitney test. Multivariate logistic regression analysis was performed to evaluate the association between the Core-Knot technique and postoperative aortic regurgitation after controlling for possible confounder. The multivariate logistic regression included tying technique, age, sex and other variables that may be associated with postoperative aortic regurgitation. The Hosmer and Lemeshow test was used to evaluate the goodness of fit of the logistic regression model. Postoperative mean gradient and cross-clamp time were natural log-transformed. Multivariate linear regressions were used to evaluate association between these variables and the tying technique after controlling for possible confounder. The multivariate linear regressions included the same variables as the logistic regression. The linear regressions were evaluated to meet the assumptions (linear relationship, normal distribution of the residuals, no multicollinearity, and homoscedasticity). A 2-tailed *P* <.05 was considered statistically significant. Analyses were performed with SPSS (IBM Corp., Released 2016, IBM SPSS Statistics for Windows, Version 24.0; Armonk, NY).

## 3 Results

In all, 94 males and 25 females with a median age of 73 years (IQR 65–78) were included in the study. Of them, 31.4% had previous percutaneous coronary angioplasty (PCI) or coronary artery bypass grafting (CABG), 20.5% had significant (≥mild) aortic regurgitation, 21.8% had some degree of mitral regurgitation (trace-mild), and 18.6% mild tricuspid regurgitation.

Aong the patents, 75 (63%) underwent the procedure using Core-Knot technique and 44 (37%) using manually tied knots. Most preoperative patient demographic characteristics were similar between groups. However, Core-Knot patients had higher preoperative aortic valve area (median 0.8 vs 0.7 cm^2^; *P* = .011) and higher ejection fraction (median 63% vs 60%; *P* = .011; Table 1). Preoperative mean gradient and occurrences of preoperative aortic regurgitation, and mitral and tricuspid regurgitation were not significantly different between groups (Table 1).

**Table 1:**
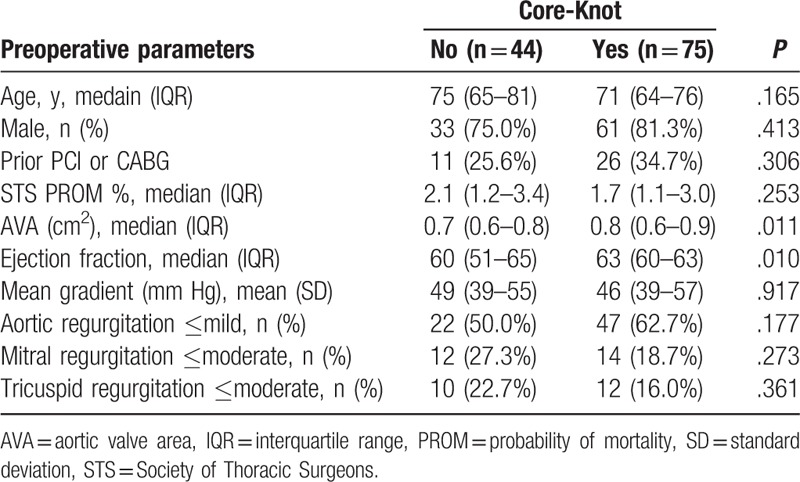
Preoperative patient characteristics.

In both surgical methods, there were no significant changes in mitral or tricuspid regurgitation. Nevertheless, patients who underwent SAVR using Core-Knot did not have new postoperative aortic regurgitation, whereas no significant change between pre and postoperative aortic regurgitation was noted when using manually tied knots (Table 3).

**Table 3:**
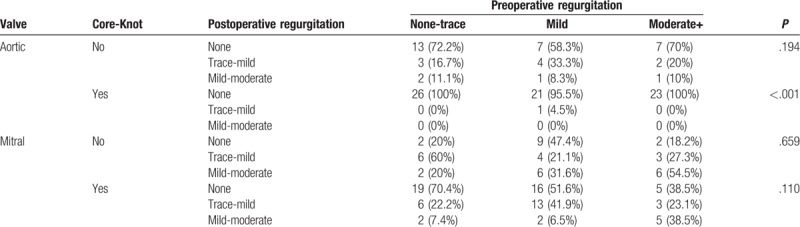
Aortic and mitral regurgitation before and after aortic valve replacement.

Thirty- day mortality was 0 for the whole group. The rates of early adverse events (including all cardiac, neurologic, and renal complications) and the immediate postprocedure echo findings were similar in the 2 groups (Table 2).

**Table 2:**
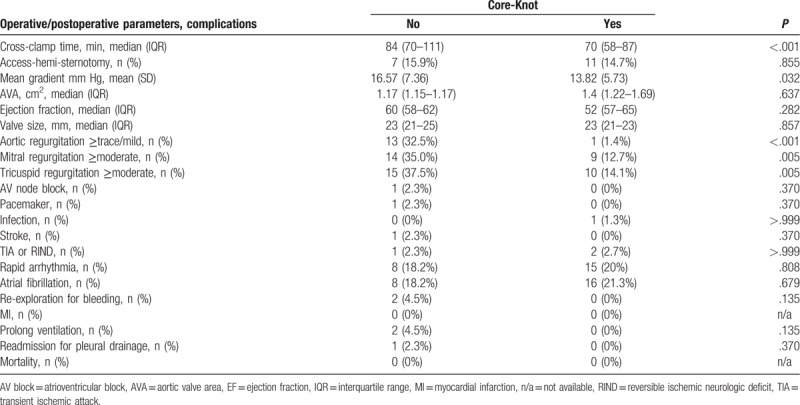
Operative and postoperative parameters and complications.

The use of Core-Knot was associated with reduced aortic cross-clamp time (median 70 vs 84 minutes; *P* < .001)), reduced postoperative mean gradients (13.82 vs 16.57; *P* = .032), and reduced rate of postoperative aortic regurgitation (1.4% vs 32.5%; *P* < .001; Table 2). In multivariate analysis, Core-Knot was associated with 19.4% shorter cross-clamp time (*P* < .001; Table 4) and 22% lower postoperative mean gradients (*P* = .007; Table 5). Core-Knot was also associated in multivariate analysis with lower risk for postoperative aortic regurgitation (*P* < .001; Table 6). Older age and bigger valve size were other predictors of reduced postoperative mean gradients, whereas preoperative ejection fraction was associated with higher postoperative mean gradients (Table 5).

**Table 4:**
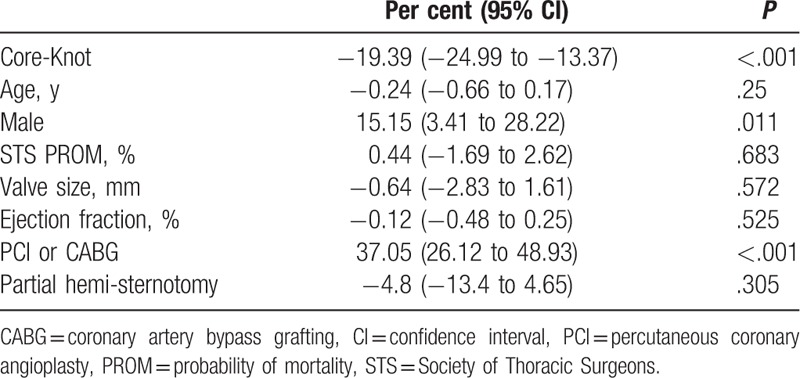
Independent predictors for cross-clamp time.

**Table 5:**
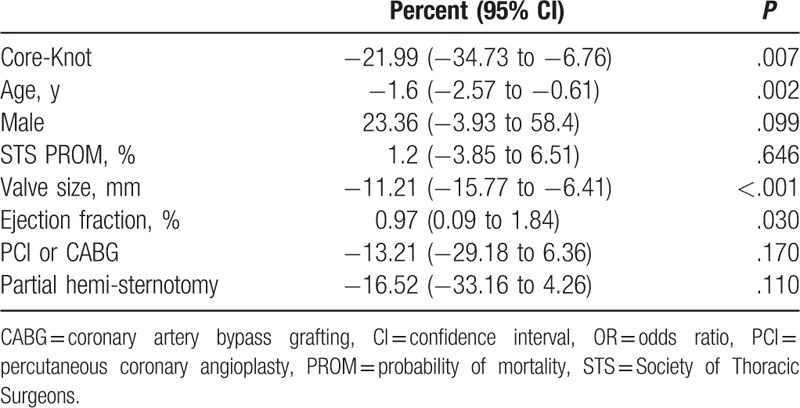
Independent predictors for postoperative mean gradient.

**Table 6:**
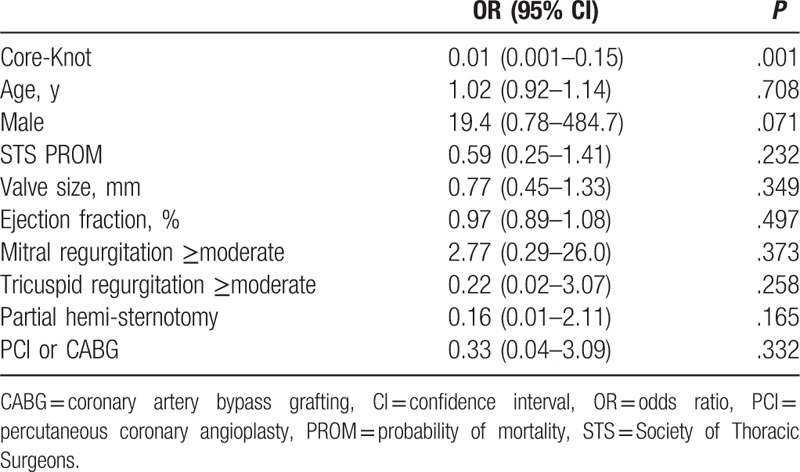
Independent predictors for postoperative aortic regurgitation.

## 4 Discussion

Our study compared 3-year (January, 2014 to December, 2016) early outcomes and characteristics of patients with aortic stenosis who underwent SAVR using the Core-Knot devise to those of SAVR patients operated using manually tied knots.

The potential importance of demonstrating an equal or better performance of a device such as the Core-Knot lies in the potential use of a device in minimally invasive cardiac surgery procedures.

The importance of performing a minimally invasive heart surgery procedure is derived out of 2 major goals and processes that have been challenging the surgical community during the past 2 decades:

1. The emerging role of transcatheter devices in treatment of structural heart disease.
2. The need to reduce the surgical burden off heart valve surgery patients.

Over the past decade, treatment options for structural heart disease and cardiac valve surgery are being redefined, due to emerging new technologies and the establishment of safety and efficacy of transcatheter devices, for certain patient subpopulations. On the contrary, other patient subgroups would still benefit from having minimally invasive heart valve surgery.

For the latter group, it is our duty as heart surgeons, to keep striving for relief of the surgical burden while keeping safety and efficacy at uncompromised levels. By introducing devices such as the Core-Knot, we do just that.

The main findings in this report are the significant reduction of aortic cross-clamp time, significant reduction of postoperative mean aortic valve gradients, and the elimination of postoperative aortic regurgitation associated with the use of Core-Knot.

Aortic stenosis is the most common clinically significant form of valvular defect in adults.^[6]^ Today, with modern myocardial preservation techniques, the average cross-clamp time required to replace a straight forward valve in patients with aortic stenosis is well within the safety limits, and were correlated to postoperative recovery times.^[10]^ However, in many cases, with combined aortic valve and CABG or combination of aortic and other valve replacement, longer ischemic times are required. In those operations, the reduction of cross-clamp time with the use of Core-Knot might be important. Reduction of cross-clamp time is also important in minimally invasive SAVR that often requires longer ischemic time.^[11]^

It has already been established that transvalvular gradients of TAVR bioprosthetic valves are lower than those of surgical valves. The lower gradients are associated with greater valve areas,^[12]^ enabling implantation of larger prosthesis with better hemodynamic function. From our study results, we might carefully question whether larger prosthesis and similar hemodynamic function may be achieved with the use of the Core-Knot in SAVR patients, and, due to the open surgical approach, better hemodynamic function is achieved without the risk of postoperative aortic regurgitation due to the paravalvular leaks associated with implantation of TAVR prosthesis^[13]^ and without the risks of conductive disturbances requiring pacemaker or vascular complications described after TAVRs.^[14]^

At this point, we do not have a sufficiently reasonable explanation for these results, although one might imagine that by causing a firmer attachment between the native aortic annulus and the prosthetic valve sewing ring with an automated fastener, we might be “opening” the left ventricular outflow tract a bit more than with manually tied knots. To clarify these results and assumption, a prospective randomized study using advanced imaging studies will be necessary.

### 4.1 Limitations

This study is a retrospective study that focuses on the comparison of early postoperative outcomes. Further studies are required with larger number of SAVR patients and surgeons and longer follow-up.

In conclusion, comparing to hand tied knots; the use of Core-Knot in SAVR reduces operative time, reduces postoperative mean gradients, and reduces the presence of any postoperative aortic regurgitation, even that regarded as trace on postoperative TEE.

## Author contributions

**Conceptualization:** Dan Loberman, Paul Pirundini, Tomer Ziv-Baran.

**Data curation:** Dan Loberman, Farhang Yazdchi, Paul Pirundini.

**Formal analysis:** Dan Loberman, Rephael Mohr, Paul Pirundini, Tomer Ziv-Baran.

**Investigation:** Dan Loberman, Daniel Rinewalt, Paul Pirundini, Tomer Ziv-Baran.

**Methodology:** Dan Loberman, Daniel Rinewalt, Paul Pirundini, Tomer Ziv-Baran.

**Project administration:** Dan Loberman, Daniel Rinewalt, Paul Pirundini.

**Resources:** Dan Loberman, Farhang Yazdchi, Daniel Rinewalt, Paul Pirundini, Tomer Ziv-Baran.

**Software:** Rephael Mohr, Tomer Ziv-Baran.

**Supervision:** Dan Loberman, Daniel Rinewalt.

**Validation:** Dan Loberman, Rephael Mohr, Daniel Rinewalt.

**Visualization:** Farhang Yazdchi, Daniel Rinewalt.

**Writing – original draft:** Dan Loberman, Farhang Yazdchi.

**Writing – review & editing:** Dan Loberman, Rephael Mohr, Farhang Yazdchi, Daniel Rinewalt, Paul Pirundini, Tomer Ziv-Baran.

## Supplementary Material

Supplemental Digital Content

## Supplementary Material

Supplemental Digital Content
